# Relationship Between Children’s Enjoyment, User Experience Satisfaction, and Learning in a Serious Video Game for Nutrition Education: Empirical Pilot Study

**DOI:** 10.2196/21813

**Published:** 2020-09-17

**Authors:** Ismael Edrein Espinosa-Curiel, Edgar Efrén Pozas-Bogarin, Juan Martínez-Miranda, Humberto Pérez-Espinosa

**Affiliations:** 1 Centro de Investigación Científica y de Educación Superior de Ensenada Unidad de Transferencia Tecnológica Tepic Tepic, Nayarit Mexico; 2 Consejo Nacional de Ciencia y Tecnología, Centro de Investigación Científica y de Educación Superior de Ensenada, Unidad de Transferencia Tecnológica Tepic Tepic, Nayarit Mexico

**Keywords:** Serious video games, children, enjoyment, user experience, satisfaction, game-based learning, nutrition, serious game, pilot study

## Abstract

**Background:**

The design and use of serious video games for children have increased in recent years. To maximize the effects of these games, it is essential to understand the children’s experiences through playing. Previous studies identified that enjoyment and user experience satisfaction of the players are principal factors that can influence the success of serious video games and the learning of their players. However, research about the relationship between enjoyment and user experience satisfaction with learning in children 8 to 10 years old is sparse.

**Objective:**

We examined the relationship of enjoyment and user experience satisfaction with the learning of children aged 8 to 10 years while playing a serious video game for health, FoodRateMaster. This serious video game teaches children about the characteristics of healthy and unhealthy foods and how to identify them in their environment.

**Methods:**

Children aged 8 to 10 years were recruited from a primary school in Mexico. Participants completed 12 individual gaming sessions with FoodRateMaster in 6 weeks. A food knowledge questionnaire was administered before and after game play to assess the players’ food knowledge. In addition, after the gaming sessions, the children’s enjoyment and user experience satisfaction were evaluated using the EGameFlow questionnaire and the Game User Experience Satisfaction Scale (GUESS) questionnaire.

**Results:**

We found significant positive associations for children’s (n=60) posttest knowledge with enjoyment (r_58_=0.36, *P*=.005) and user experience satisfaction (r_58_=0.27, *P*=.04). The children’s posttest knowledge scores were also positively correlated with challenge (r_58_=0.38, *P*=.003), knowledge improvement (r_58_=0.38, *P*=.003), and goal clarity (r_58_=0.29, *P*=.02) EGameFlow subscales and with narrative (r_58_=0.35, *P*=.006), creative freedom (r_58_=0.26, *P*=.04), and visual esthetics (r_58_=0.32, *P*=.01) GUESS subscales. Regression analysis indicated that the EGameFlow (F_7,52_=2.74, *P*=.02, R^2^=0.27) and the GUESS (F_8,51_=2.20, *P*=.04, R^2^=0.26) ratings significantly predicted the children’s posttest knowledge scores. EGameFlow challenge (β=0.40, *t*_52_=2.17, *P*=.04) and knowledge improvement (β=0.29, *t*_52_=2.06, *P*=.04) subscales significantly contributed to predicting children’s learning. None of the GUESS subscales significantly contributed to predicting children’s learning.

**Conclusions:**

The findings of this study suggest that both enjoyment and user experience satisfaction for children aged 8 to 10 years were positively correlated with their learning and that were significant predictors of it. Challenge, knowledge improvement, narrative, creative freedom, and visual esthetics subscales correlated positively with children’s learning. In addition, challenge and knowledge improvement contributed to predicting their learning. These results are relevant to consider during the design stages of serious games developed for young children’s learning purposes.

## Introduction

### Serious Video Games for Children

Serious video games are interactive digital programs or apps that have a goal beyond entertainment or fun. This type of video game is designed for promoting and encouraging a broad range of purposes such as education, training, skill development, knowledge acquisition, or even behavior and attitude changes [1,2]. The primary rationale of using video games for serious purposes is to take advantage of their ability to motivate, provide enjoyment, and engage players [1]. In addition, their versatility allows them to be used as tools in a broad spectrum of domains such as the military, government, educational, corporate, enterprise, tourism, and health [3]. Most serious games have reported positive outcomes, and in general, they are more effective than conventional instructional methods [4-7].

The development and use of serious video games designed for children have increased over the past decade [8-11]. Researchers have encouraged their use in children, since children (regardless of age, sex, and background) are interested in video games [9], and because video games allow them to practice different skills in a safe environment. In addition, video games can be used as an alternative to influence children who are having difficulties learning with traditional education methods [1]. There are currently serious video games for children for a broad set of applications, such as STEM education, obesity prevention, nutrition education, chronic diseases, psychiatry, learning disabilities, and cognitive development [9-14].

While there is a growing body of literature on the potential of serious video games in children [9-15], researchers have tended to ignore what children experience during playing sessions, and how these experiences influence the success, effectiveness, and outcomes of serious games. Having a better understanding of their experiences can help in designing more effective and engaging serious video games for children [16-20].

### Enjoyment and User Experience Satisfaction in Serious Games

Two of the best-known factors that influence the acceptance, use, and success of any video game are the players’ enjoyment and the players’ experience satisfaction [21-26]. If players do not enjoy the game or have a satisfying gaming experience, they will not play the game [27]. However, there is a lack of consensus in the video game field regarding the definitions of enjoyment and user experience satisfaction [21,24]. Moreover, as terms, they are often used interchangeably with a host of other terms, some of which overlap conceptually (eg, engagement, flow, fun, playability, and immersion) [21,24].

According to Crutzen et al [21] *enjoyment* refers to “the action or state of deriving gratification from a video game.” Enjoyment is a multidimensional concept that embraces dimensions such as concentration, goal clarity, feedback, challenge, autonomy, immersion, social interaction, knowledge improvement, competence, narrative transportation, and relevance [21,27,28]. Meanwhile, Phan et al [24] defined game user *experience satisfaction* as “the degree to which the players feel gratified with his or her experience while playing a video game.” Game user experience satisfaction involves different dimensions such as usability (or playability), narratives, creative freedom, audio and visual esthetics, and personal gratification [23,24,26].

The dimensions of enjoyment and player experience satisfaction are important factors for the success and effectiveness of serious games [17,21,29] since boring or poorly designed serious video games run the risk of disappointing and eventually alienating their target audience [30]. Previous studies have identified that factors such as technological capacity, esthetic presentation, game design elements, and fun factors (eg, characters, dialogues, and humor) influence the acceptability of serious games [17]. In addition, factors such as game goals, freedom, narrative, interaction, challenge, sensation, feedback, level of fun, usability, and mystery are critical for the effectiveness of serious games [29,31]. These factors also can influence the mood [19], engagement [21,32], satisfaction [33-35], and performance [34,36] of players. Serious video games can provide excellent levels of usability [35,37] and enjoyment [38] in comparison with entertainment video games [17,39].

### Enjoyment, User Experience Satisfaction, and Learning in Serious Games

The studies [20,40-44] that describe the relationship between enjoyment and user experience satisfaction with the learning of players in serious games show contradictory conclusions. While some studies have stated that enjoyment could be perceived as an outcome opposite to learning in serious games [40,41], other studies noted that enjoyment and user experience satisfaction could positively impact players’ motivation to learn and the learning outcomes. In particular, attributes such as fantasy, representation, sensory stimuli, mystery, control, assessment, narrative, realism, adaptivity, interaction, feedback, debriefing, graphics, fun, rules, and goals could support players’ learning [42], influence players’ cognitive engagement and motivation [20,42,43], and impact players’ learning outcomes [20,44].

Despite enjoyment and user experience being essential concepts that are important in fostering learning in serious games, surprisingly few studies [45-50] have measured the dimensions of enjoyment and user experience satisfaction in the serious game context and analyzed how it relates to the learning of players. Giannakos [45], in a study where middle school students played a math game, identified that players’ enjoyment had a significant relationship with learning performance. Hamari et al [46], in a study where high school students played an optics game and an engineering dynamics game, identified that engagement had a positive effect on learning, challenge affected learning, and perceived challenge was a strong predictor of learning outcomes. Conversely, they determined that immersion did not have a significant effect on learning. Fokides et al [47], in a study where university students played a history game and a math game, identified that enjoyment, goal clarity, realism, and narration adequacy were the most influential factors shaping user views for serious game learning effectiveness; conversely, they determined that presence, ease of use, and motivation had a marginal effect. Iten and Petko [48], in a study where primary school students (aged 10 to 13 years old) played a game to develop internet skills, identified that enjoyment could encourage players to learn, but it had a small influence on learning gains. In a more recent study with students aged 9 to 12 years old, Iten and Petko [49] identified that self-reported cognitive learning gains were positively correlated with enjoyment. Ebrahimzadeth and Alavi [50], in a study where high school students played a commercial strategy game, identified that enjoyment significantly predicted players’ vocabulary learning.

While there is a growing body of literature on the importance of enjoyment and experience satisfaction for players when playing serious video games and their relationship with players learning, currently, there is a dearth of studies that investigated the relationship between these concepts and the learning of young children prior to adolescence (aged between 8 to 10 years) in the context of serious games. Most abovementioned studies [45-50] were evaluated with adolescents (between 10 to 19 years old) [45-49] or young adults [50], and those that included children also included adolescents [48,49], despite the differences in their intellectual and emotional growth [51,52]. The importance of enjoyment and user experience satisfaction and the influence of these concepts on learning could be different for children than for adolescents or young adults [19].

### Objective

This study responded to this research gap by examining the relationship between learning, user experience satisfaction, and enjoyment in children aged between 8 to 10 years when playing a serious video game.

### Hypotheses

Hypothesis 1: Enjoyment relates positively to children’s learning.

Hypothesis 2: Enjoyment influences children’s learning.

Hypothesis 3: User experience satisfaction relates positively to children’s learning.

Hypothesis 4: User experience satisfaction influences children’s learning.

## Methods

### Overview

This research is part of a comprehensive project to understand the effectiveness of serious video games in children. The video game FoodRateMaster and the research procedure used to obtain the learning data have previously been described in detail [53].

### FoodRateMaster

A multidisciplinary team developed FoodRateMaster through an extensive formative research process. FoodRateMaster was designed specifically to teach young children about the suggested ranges for food nutrients to help them determine if they should decrease or maintain their intake of certain foods [53].

**Figure 1:**
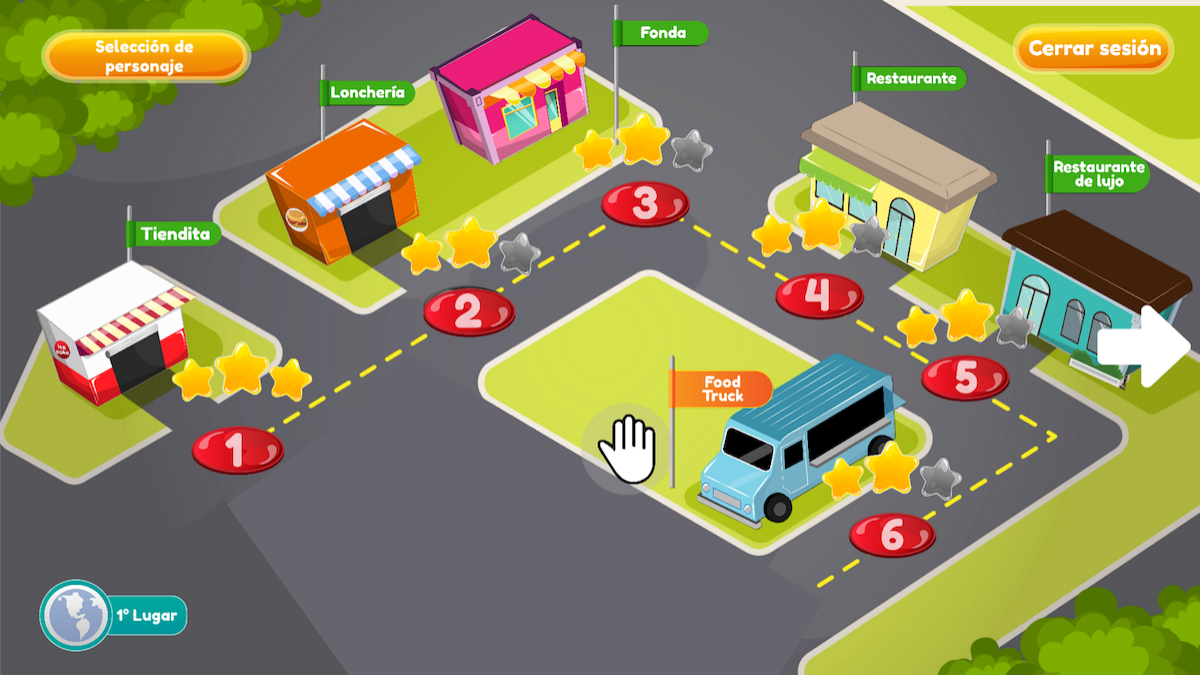
FoodRateMaster scenario map.

**Figure 2:**
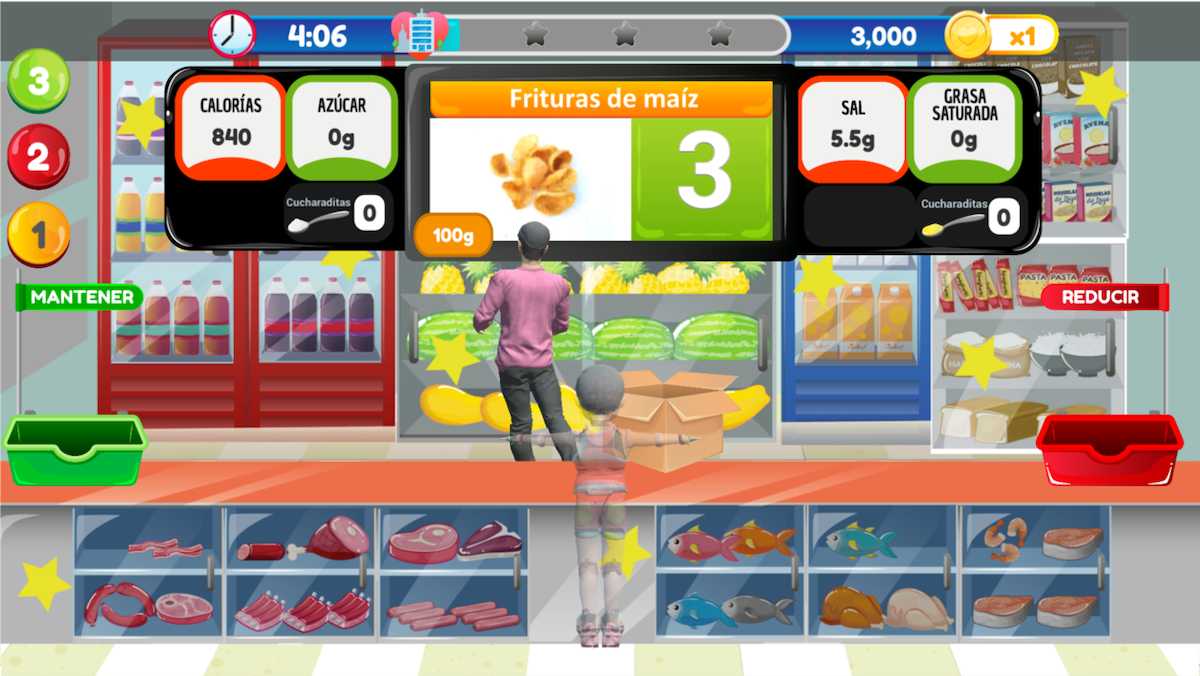
FoodRateMaster game scenario.

**Figure 3:**
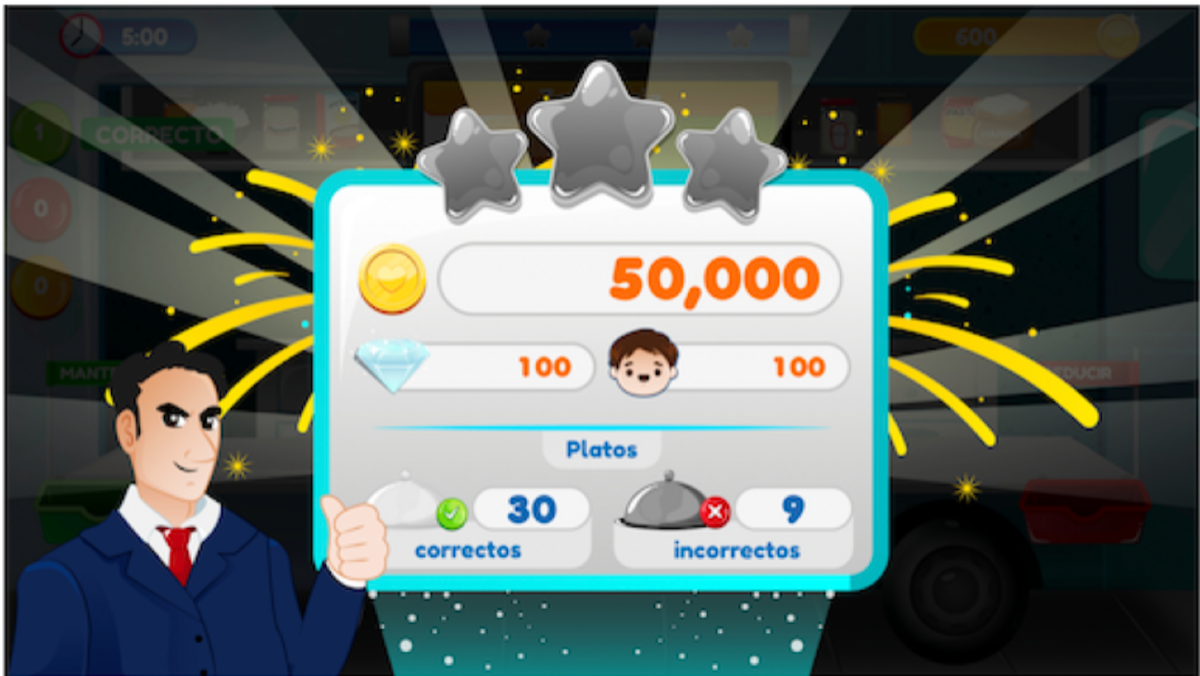
FoodRateMaster level results screen.

FoodRateMaster has 6 levels (Figure 1) that replicate a real food establishment (eg, a food truck, a restaurant, or a grocery store). In each scenario (Figure 2), an evil chef prepares a menu; players, based on the food's nutritional information, determine whether food can be consumed (healthy) or whether its consumption must be reduced (unhealthy). Players use their avatar to take healthy foods (with green labels) and put them into the “keep basket” (green basket) and take unhealthy foods (yellow and red labels) and put them into the “reduce basket” (red basket). Players move the avatar to classify food and avoid obstacles. Maintaining healthy food or reducing unhealthy food allows them to maintain a healthy lifestyle and therefore win points. Additionally, players earn extra points when they correctly replace unhealthy food with healthy food. Otherwise, if players make too many errors, then they do not score enough points to unlock subsequent levels. Finally, a results screen specifies points earned, incorrect food classifications, and position in the global ranking of players (Figure 3).

**Figure 4:**
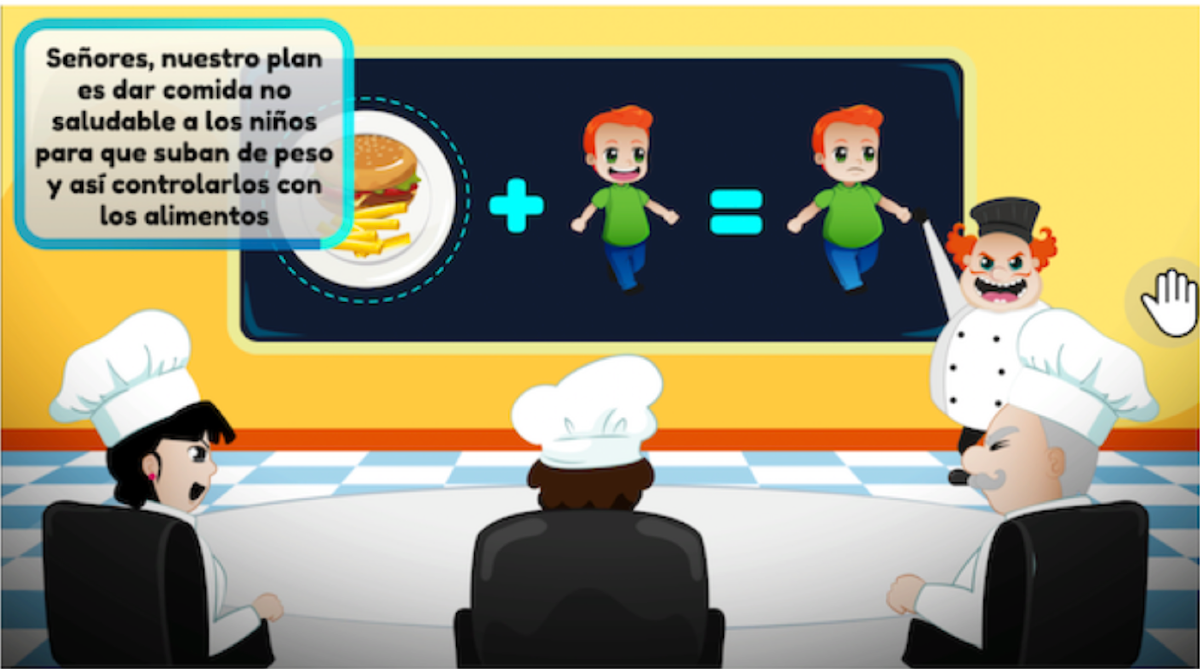
Story of FoodRateMaster game.

**Figure 5:**
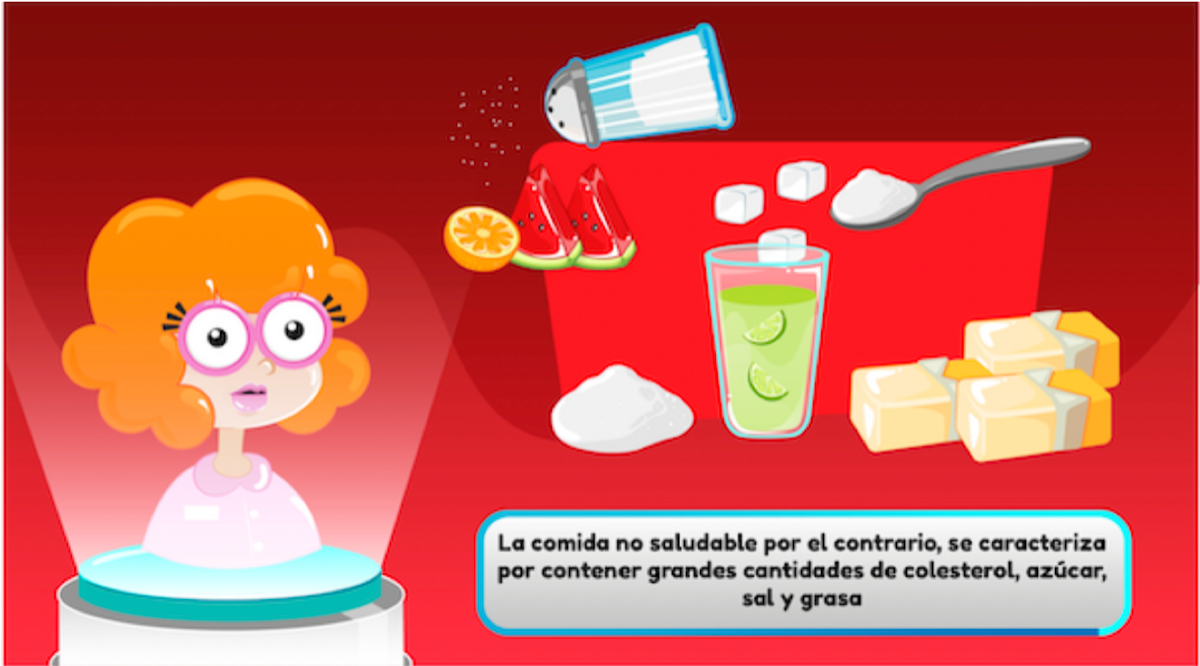
FoodRateMaster explanation of unhealthy food.

Several key elements of FoodRateMaster were designed to encourage enjoyment and produce a satisfying user experience [53]: (1) A history of why the players needs to complete the game mission is given using 2 characters: evil chefs and kids who need to be more selective about the food they eat (Figure 4). (2) Narrative, feedback mechanisms, and attractive audio and visual elements were used to encourage the players’ immersion, concentration, and enjoyment. (3) A graded task mechanism with a cumulative difficulty curve through different levels was used to adjust the challenge and encourage players’ fun through the end of the game (Figure 1). (4) Learning strategies, such as behavioral repetition and substitution (Figure 2) and cognitive restructuring were used to guide knowledge improvement (Figure 5). In addition, in FoodRateMaster, children have to use their creativity and curiosity to figure out how to use the information on nutrition labels and nutrition traffic lights to make better decisions about what foods to eat. An example of these strategies is to use the associations between foods to facilitate and speed up their decision making. (5) Emotional scenes of children were used (eg, when they consume an unhealthy meal, gain weight and can suffer from teasing and bullying). (6) A scoring system and ranking board assessed players’ gaming performances.

### Participant Recruitment

Children aged to 10 years (n=62) at a primary school (grades 3, 4, and 5) in Mexico were invited to participate in the pilot study. We conducted a presentation of FoodRateMaster to the children to explain its purpose, characteristics, elements, and how to play it. We obtained written consent from the parents of the children who were interested in participating. Participation was voluntary, and no additional efforts for recruitment were made. The institutional review board of the *Centro de Investigacion Cientifica y de Educacion Superior de Ensenada* (Ensenada Center for Scientific Research and Higher Education) approved this study.

### Measures

We used the following 3 questionnaires to collect data: a food knowledge questionnaire [53], the EGameFlow questionnaire [54], and the Game User Experience Satisfaction Scale (GUESS) questionnaire [24].

The food knowledge questionnaire [53] was developed by a multidisciplinary team based on 90 frequently consumed foods in Mexico (49 healthy and 41 unhealthy). A 3-point response format is used to evaluate each food—*healthy* (maintain or increase intake), *unhealthy* (reduce intake), or *I do not know*. The score is the total number of questions answered correctly, with a maximum score of 90.

The EGameFlow questionnaire [54] is a valid and reliable tool for evaluating the level of enjoyment provided by electronic learning games to their user. This scale includes 42 items in 8 subscales [27,54]: *concentration*—games should require concentration, and players should be able to concentrate on the game; *goal clarity*—games should provide players with clear goals at appropriate times; *feedback*—players must receive appropriate feedback about their actions and their progress toward their goals at appropriate times; *challenge*—games should be sufficiently challenging and match players’ skill level; *autonomy*—players should feel a sense of control over their actions in the game; *immersion*—players should experience deep but effortless involvement in the game; *social interaction*—games should support and create opportunities for social interaction; *knowledge improvement*—games support and motivate players to acquire, integrate, and apply the knowledge that is taught. The GUESS questionnaire [24] is a valid and reliable tool for evaluating user satisfaction of different video game types by a variety of users with 55 items in 9 subscales: *usability* (or *playability*)—games can be played easily with clear goals or objectives in mind and with minimum interference from the interfaces and controls; *narratives*—the story aspect of the game and its ability to capture players’ interest and shape players’ emotions; *play engrossment*—games should hold players’ attention and interest; *enjoyment*—the pleasure of players that results from playing the game; *creative freedom*—games should foster players’ creativity and curiosity; *audio esthetics*—the auditory aspects of the game (eg, sound effects) and how much they enrich the gaming experience; *personal gratification*—players’ sense of accomplishment and the desire to succeed and continue playing the game; *social connectivity*—games should facilitate social connections between players; *visual esthetics*—the graphics of the game and how attractive they appear to players. The EGameFlow scale is one of the most widely used questionnaires to evaluate the enjoyment of serious games [55].

We used all subscales for both scales except EGameFlow social interaction and GUESS social connectivity because FoodRateMaster does not include social features. According to Phan et al [24] GUESS subscales that are not applicable (ie, features not included in the video game being evaluated) can be omitted. In both questionnaires, a 5-point Likert-type response format ranging from *strongly disagree* to *strongly agree* was used to measure each item. We used only 5 points because having additional points on the response scale provides little additional utility [56] and can influence the item values and the psychological distance [57]. In addition, children can discriminate among 5 response options and do not tend toward the neutral point [58].

### Data Collection

In the pretest phase, the participants were assigned unique identifiers and completed the food knowledge questionnaire. During the next 45 days, the children conducted 12 game sessions of at least 15 minutes each with FoodRateMaster (playing time: mean 3.5 hours, SD 0.8). In the posttest phase (the day after the last game session), children completed the food knowledge questionnaire (again), the EGameFlow questionnaire [54], and the GUESS questionnaire [24]. The children took approximately 15 minutes to complete each questionnaire. Initially, we conducted a pilot test of these questionnaires and identified that children did not have any problem when answering them.

### Data Analysis

Data analysis was carried out using the SPSS statistical software (version 26; IBM Corp). In all analyzes, the statistical significance was 5% (*P*<.05). A paired two-tailed *t* test was conducted to examine the difference between pretest and posttest knowledge scores. Pearson correlation was conducted to examine the relationships of enjoyment and user experience satisfaction, including their subscales, with posttest knowledge scores. We also performed standard multiple regression on EGameFlow and the GUESS scores to identify if their subscales could predict the results of the posttest children’s knowledge and to examine their correlations. Separate multiple linear regressions were calculated to predict the posttest children’s knowledge based on (1) EGameFlow and (2) GUESS subscales. Preliminary analyses were performed to ensure that the assumptions of linear regression analysis were not violated (the criteria are included in parentheses) [59,60]: linearity, normality, nonzero variance (variance >0), no data outliners (standardized residual values for each participant from −3.29 to 3.29), collinearity (for all variables, variance inflation factor is <10 or tolerance >0.1), and independent errors (Durbin-Watson values from 1.5 to 2.5).

## Results

Data from 2 children were excluded from the analysis (ie, these 2 students did not complete all the questionnaires); thus, the study completion rate was 97% (60/62): 29 girls and 31 boys (age: mean 9, SD 0.8 years).

The mean pretest and posttest knowledge scores were 56.95 (SD 10.71, range 30-75) and 67.88 (SD 10.71, range 30-85), respectively. In addition, the mean enjoyment and user experience satisfaction scores were 4.16 (SD 0.55, range 2.28-5.00) and 4.29 (SD 0.54, range 2.79-5.00), respectively. Knowledge scores were significantly lower in the pretest than in the posttest (mean difference −11.12, SD 8.27; t_59_=−10.41, *P*<.001). Thus, children increased their nutritional knowledge after game play.

We found associations (Table 1) between posttest knowledge scores and enjoyment (EGameFlow scale: *r*_58_=0.36, *P*=.005), as well as between posttest knowledge scores and user experience satisfaction (GUESS scale: *r*_58_=0.34, *P*=.008). Additionally, we found associations between posttest knowledge and the following EGameFlow subscales: challenge (*r*_58_=0.38, *P*=.003), knowledge improvement (*r*_58_=0.38, *P*=.003), and goal clarity (*r*_58_=0.29, *P*=.02). Also, associations were found between posttest knowledge and the following GUESS subscales: narrative (*r*_58_=0.35, *P*=.006), creative freedom (*r*_58_=0.26, *P*=.04), and visual esthetics (*r*_58_=0.32, *P*=.01).

Both models fulfilled all the assumptions of linear regression analysis. The regression results indicated that the model based on the EGameFlow scale explained 27% of the variance (large effect [61]) and that the model was a significant predictor of children’s learning (*F*_7,52_=2.74, *P*=.02). Table 2 describes the contribution of each subscale to the total explained variance; only 2 subscales had significant contributions, namely, challenge (β=0.40, t_52_=2.17, *P*=.04) and knowledge improvement (β=.29, t_52_=2.06, *P*=.04). Meanwhile, the regression results indicated that the model based on the GUESS scale explained 26% of the variance (large effect) and that the model was a significant predictor of children’s learning (*F*_8,51_=2.20, *P*=.04). However, as shown in Table 2, none of the subscales had a significant contribution.

**Table 1:** Bilateral correlations of children’s posttest knowledge with EGameFlow and Guess scales and subscales.

EGameFlow and GUESS subscales			Posttest knowledge score		
			*r*		*P* value (bilateral)
**EGameFlow (Enjoyment^a^)**			**0.36**		**.005** ^b^
	Concentration	0.09		.49	
	Goal clarity	0.29		.02^b^	
	Feedback	0.17		.19	
	Challenge	0.38		.003^b^	
	Autonomy	0.01		.93	
	Immersion	0.06		.65	
	Knowledge improvement	0.38		.003^b^	
**GUESS (User experience satisfaction^c^)**			**0.27**		**.04** ^b^
	Usability/playability	0.21		.11	
	Narratives	0.35		.006^b^	
	Play engrossment	−0.15		.25	
	Enjoyment	0.09		.47	
	Creative freedom	0.26		.04^b^	
	Audio esthetics	0.09		.45	
	Personal gratification	0.15		.24	
	Visual esthetics	0.32		.01^b^	

^a^Enjoyment as a whole (mean of subscale scores).

^b^*P*<.05.

^c^User experience satisfaction as a whole (mean of subscale scores).

**Table 2:** Model coefficients for EGameFlow and GUESS subscale ratings with posttest knowledge as the dependent variable.

EGameFlow and GUESS subscales		β	*t* value^a^	*P* value
**EGameFlow scale—Enjoyment**				
	Concentration	0.03	0.24	.81
	Goal clarity	0.14	0.89	.38
	Feedback	0.13	−0.72	.48
	Challenge	0.40	2.17	.04^b^
	Autonomy	0.08	−0.58	.56
	Immersion	0.19	−1.30	.20
	Knowledge improvement	0.29	2.06	.04^b^
**GUESS scale—User experience satisfaction**				
	Usability/playability	0.30	1.41	.16
	Narratives	0.26	1.69	.10
	Play engrossment	−0.23	−1.79	.08
	Enjoyment	0.02	0.12	.91
	Creative Freedom	0.22	1.42	.16
	Audio esthetics	−0.34	−1.93	.06
	Personal gratification	−0.21	−0.99	.32
	Visual esthetics	0.22	1.19	.24

^a^Model *df*=52 for EGameFlow and *df*=51 for GUESS.

^b^*P*<.05.

## Discussion

### Principal Results

This study investigated the relationships of enjoyment and user experience satisfaction with children’s learning while playing a serious video game. As far as we know, this is the first paper analyzing these relationships in children aged 8 to 10 years. Overall, the study’s findings suggest that serious games can provide enjoyment and satisfactory user experiences to children, as demonstrated by the levels of enjoyment and user experience satisfaction. The results specifically showed significant associations for children’s enjoyment and user experience satisfaction with their learning when playing serious video games. In addition, the ratings given by children on subscales of the questionnaires used to assess enjoyment and user experience satisfaction were significant predictors of their learning. Challenge and knowledge improvement (EGameFlow) were correlated with children’s learning and contributed to predicting it. Meanwhile, narrative, creative freedom, and visual esthetics (GUESS) were correlated with children’s learning; however, did not contribute toward predicting learning.

### Comparison With Prior Work

Associating the findings with the results of previous research is a difficult task, given that our study examined the influence of enjoyment and user experience on the learning of young children (aged between 8 to 10 years old), in contrast to the existing studies that involved adolescents or young adults [45-47]. Even so, the findings that enjoyment and user experience were associated with learning and can help to predict player learning provide further support to findings in previous studies [45-47,49,50]. In addition, this is the first study (as far as we know) that analyzed the relationship of these factors in the context of a serious game for health.

### Enjoyment and Game-based Learning

Hypotheses 1 and 2, which emerged from the expectation that enjoyment positively correlates to children’s learning and can predict it, were confirmed (hypothesis 1: *r*_58_=0.36, *P*=.005; hypothesis 2: *F*_7,52_=2.74, *P*=.02). These results are in line with previous findings that enjoyment had a significant relationship with learning performance [45-47,49] and that enjoyment can predict learning [50], and in addition, that this factor significantly contributed to the effectiveness of serious games [4,17].

Using the EGameFlow scale, challenge and knowledge improvement were found to correlate with and contribute to predicting children’s learning. Regarding challenge, this finding was anticipated since the relationship between challenge and skill determines players’ engagement level during gameplay [62]. Thus, the optimal amount of difficulty should match user’s abilities to the skills required to accomplish each goal [27]. This result supports the findings of several studies that have indicated that challenge is a critical factor in the success of serious games [20,29,34,43] and related to players’ learning [46,50]. Regarding knowledge improvement, serious games should incorporate strategies to provide knowledge that players need to learn and integrate to fulfill the goals of the game [54]. Our results supports those of Ebrahimzadeth and Alavi [50], who identified that knowledge improvement is positively correlated with players’ learning. In addition, goal clarity correlated with children’s learning. This results add support to the findings of studies [43,47] that stated that goal clarity enhances the engagement levels of players and influences learning effectiveness. However, this result contrasts with the findings of other studies [46,47,50] that identified that goal clarity does not influence the players’ learning.

Concentration (*P*=.49), feedback (*P*=.19), autonomy (*P*=.93), and immersion (*P*=.65) were not correlated with children’s learning, nor did they predicting it. These results may be related to how children perceive the feedback of serious games, their need for autonomy and cognition, and their sensation seeking [43]. This result gives further support to those of previous studies [46,47,50], noting that concentration, feedback and immersion do not influence players’ learning. However, this result contrasts with findings in other studies stating that concentration is essential for learning in serious games [18,27], that feedback influences the learning of players [18,20,36,43,44], and that autonomy is an important factor in the effectiveness of serious games [20,29,43] and is correlated with players’ learning [50]. Finally, the actual impact of immersion on learning outcomes seems more complicated in the use of video games for educational purposes [63]. While some studies [31,50,64] have noted that immersion is an important factor in the effectiveness of serious games [31] and is related to players’ learning [50,64], other studies [63,65] have identified that immersion can limit players’ learning because players can ignore the educational targets of video games due to the increased cognitive load.

### User Experience Satisfaction and Game-based Learning

Hypotheses 3 and 4, emerging from the expectation that user experience satisfaction positively correlates to and can predict children’s learning were confirmed by the data analysis (hypothesis 3: *r*_58_=0.27, *P*=.04; hypothesis 4: *F*_8,51_=2.20, *P*=.04). Comparing these findings with those of previous research is difficult due to most existing studies focusing on using the GUESS scale to evaluate serious games (eg, [66,67]) and (as far we know) no previous studies have addressed the relationship between the GUESS scale and subscales with players’ learning.

Using GUESS, narrative, creative freedom, and visual esthetics correlated with children’s learning, but none contributed toward predicting children’s learning. Narration facilitates situated cognition by immersing players in a setting that frames knowledge [68]. Our results support those of studies that noted the impact of a narrative on learning [17,21,29,43,44,69] and that identified that a narrative is positively correlated with players’ learning [47,50]. Regarding creative freedom, playing is a creative process by itself that can foster new and novel associations between ideas, objects, and behaviors [70]. In this way, serious games provide a safe environment for active, critical, and creative learning, allowing users to explore skills, methods, and concepts [71]. Previous studies [72,73] have reported the usefulness of serious games for ideation; however, no previous studies have analyzed the correlation between creative freedom and players’ learning, as far as we know. In addition, our results give further support to those of previous studies noting the influence of visual esthetics on the acceptability of serious games and the learning of players [17,20,29,33,42,44] and reporting a statistical association of the esthetic presentation of the game upon the learning outcome [74].

In this study, usability (or playability) (*P*=.11), play engrossment (*P*=.25), enjoyment (*P*=.47), audio esthetics (*P*=.45), and personal gratification (*P*=.24) did not reach a statically significant correlation with children’s learning, nor did they contribute to predicting it. Our results support the results of Fokides et al [47], who found that there was no significant relationship between usability (or playability) and the learning of players and that it only influenced players’ enjoyment. However, our results are in contrast to the findings of Tolentino et al [35], who stated that serious video games should have a high level of usability. Player engrossment is similar to the immersion subscale of the EGameFlow scale, which was not associated with learning. The absence of correlation between the enjoyment subscale of the GUESS scale and children’s learning is surprising because there was a correlation between the score of the EGameFlow scale and children’s learning. We consider that this result is due to the enjoyment subscale of the GUESS scale and does not include all the factors that influence enjoyment considered in the EGameFlow scale. Finally, although previous research also noted the importance of audio esthetics [20,29,75] and personal gratification [29] to generate motivation and immersion in serious games, no previous studies have analyzed the correlation between these subscales and the learning of players, as far as we know.

### Limitations and Future Work

This study faced some limitations. First, the self-report measure of enjoyment and user experience satisfaction can be highly sensitive to the respondent’s comprehension and willingness to provide honest answers. Second, the results should be interpreted with caution since a small sample of children participated in the study. The sample size could have been larger, allowing for more confidence in the robustness of the statistical analysis and results. However, given that our main objective was to explore the relationship of children’s learning with enjoyment and user experience satisfaction, we believe that our results provide a good starting point for researchers to explore the design of serious video games for children. Third, due to space and time constraints, children who were not from the same class attended at the same time to play on the play stations. This situation might have affected the participant behaviors (ie, higher or lower performance in the game). Fourth, some students were more familiar with video games, which might have affected their performance. Fifth, the frequency and duration of the sessions were predefined. In real environments, it is common for game sessions to be longer and more frequent, which could accelerate the rate at which players become bored with the video game and can even generate video gaming burnout [76], consequently affecting learning, enjoyment, and user’ satisfaction. Sixth, the students also had a limited age range, limiting the generalizability of the findings. Finally, the children played only one serious game. To overcome these issues, we suggest similar studies be carried out with students of different age groups and levels of experience with video games, using several different serious video games, and where players can carry out their sessions at their own pace.

For future work, we plan to carry out a comprehensive study with users in a broad age range to provide generalizable results. In addition, based on the insight that the influence of enjoyment and user experience satisfaction could depend on the age of players, in a future study, we also plan to perform age-based analysis to assess the importance of age and determine its association with the relationship of enjoyment and user experience satisfaction with learning, as well as whether there is variability between different subscales among users of different ages. 

### Conclusions

In summary, the reported findings from this study can be useful to better understand the correlation and influence of enjoyment and user experience on children’s learning when playing serious games. These findings should be considered in the design stages during the development of serious video games for children and suggest that, because children’s enjoyment and user experience satisfaction are significantly associated with their learning, and enjoyment and user experience satisfaction are significant predictors of children’s learning while playing a serious video game, when serious video games are designed for children, it is essential to implement elements that foster enjoyment and user experience satisfaction. In particular, video games should be sufficiently challenging, match children's skills, and include elements supporting and motivating players to acquire and apply the learned knowledge. Clear goals, narrative, creative freedom, and visual esthetics are positively correlated with children’s learning; therefore, designers of serious video games for children should dedicate efforts to providing clear goals and producing elements that capture players’ interest, shape players’ emotions, and foster their creativity and curiosity, as well as include attractive graphical elements in the game.

## Abbreviations

GUESS: Game User Experience Satisfaction Scale
STEM: science, technology, engineering, math
